# The Bacterial Gut Microbiota of Schoolchildren from High and Low Socioeconomic Status: A Study in an Urban Area of Makassar, Indonesia

**DOI:** 10.3390/microorganisms8060961

**Published:** 2020-06-26

**Authors:** Aldian I. Amaruddin, Firdaus Hamid, Jan Pieter R. Koopman, Munawir Muhammad, Eric A.T. Brienen, Lisette van Lieshout, Anoecim R. Geelen, Sitti Wahyuni, Ed J. Kuijper, Erliyani Sartono, Maria Yazdanbakhsh, Romy D. Zwittink

**Affiliations:** 1 Department of Parasitology, Leiden University Medical Center, 2333ZA Leiden, The Netherlands; J.P.R.Koopman@lumc.nl (J.P.R.K.); E.A.T.Brienen@lumc.nl (E.A.T.B.); E.A.van_Lieshout@lumc.nl (L.v.L.); E.Sartono@lumc.nl (E.S.); M.Yazdanbakhsh@lumc.nl (M.Y.); 2 Department of Parasitology, Faculty of Medicine, Hasanuddin University, Makassar 90245, Indonesia; sittiwahyuni@gmail.com; 3 Department of Microbiology, Faculty of Medicine, Hasanuddin University, Makassar 90245, Indonesia; firdaus.hamid@gmail.com (F.H.); dr.munawirmuhammad@yahoo.co.id (M.M.); 4 Experimental Bacteriology, Department of Medical Microbiology, Leiden University Medical Center, 2333ZA Leiden, The Netherlands; A.R.Geelen@lumc.nl (A.R.G.); E.J.Kuijper@lumc.nl (E.J.K.); 5 Center for Microbiome Analyses and Therapeutics, Leiden University Medical Center, 2333ZA Leiden, The Netherlands

**Keywords:** gut microbiota, socioeconomic status, intestinal parasites, nutritional status, schoolchildren

## Abstract

To understand the relationship between the gut microbiota and the health profile of Indonesians, it is important to elucidate the characteristics of the bacterial communities that prevail in this population. To this end, we profiled the faecal bacterial community of 140 Indonesian schoolchildren in urban Makassar. The core microbiota of Indonesian schoolchildren consisted of *Bifidobacterium*, *Collinsella*, and multiple members of the *Lachnospiraceae* and *Ruminicoccaceae* families, but the relative abundance of these taxa varied greatly among children. Socioeconomic status (SES) was the main driver for differences in microbiota composition. Multiple bacterial genera were differentially abundant between high and low SES children, including *Bifidobacterium, Lactobacillus, Prevotella*, and *Escherichia-Shigella*. In addition, the microbiota of high SES children was less diverse and strongly associated with body mass index (BMI). In low SES children, helminth infection was prevalent and positively associated with *Olsenella, Enterohabdus,* *Lactobacillus*, and *Mogibacterium* abundance, while negatively associated with relative abundance of *Prevotella*. Protozoa infection was also prevalent, and positively associated with *Rikenellaceae,* while it was negatively associated with the relative abundance of *Romboutsia* and *Prevotella*. In conclusion, Indonesian schoolchildren living in urban Makassar share a core microbiota, but their microbiota varies in diversity and relative abundance of specific bacterial taxa depending on socioeconomic status, nutritional status, and intestinal parasites infection.

## 1. Introduction

In the past decades, several studies have established the role of the gut microbiota in maintaining host physiological states, including immune responses, metabolism, mental, and physical development [1]. The imbalance of gut microbiota composition may aggravate inflammation, metabolic diseases, or other health problems [2,3,4,5].

Gut microbiota composition is predominantly driven by environmental factors, such as diet, physical activity level, hygiene, disease, and medication use, instead of genetics [6,7]. Previous studies revealed notable differences in gut microbiota profiles among different ethnic and geographical areas, sometimes representing socioeconomic inequalities between groups [8,9,10,11]. A study involving European and African children has shown that the microbiota of African children is more diverse and contains more fibre-degrading, short-chain fatty acid producing bacteria than European children, attributable to differences in diet and lifestyle [12].

Indonesia is a developing country with enormous economic growth, but with great socioeconomic disparities in its population. This inequality is also reflected by the wide gap in health status between people from high and low socioeconomic status (SES). Low SES has been associated with lack of sanitation and bad hygiene and, as a consequence, higher exposure to soil-transmitted-helminths or other intestinal parasites, especially in schoolchildren. Recently, we investigated gut microbiota composition in a helminth endemic area in rural Indonesia. When comparing helminth infected with uninfected individuals, we observed no difference in bacterial composition or diversity of the gut microbiota [13]. Since there are no large disparities in lifestyle and SES in that area, these factors were not studied therein.

Given the role of the gut microbiota in health and susceptibility to diseases, it is important to investigate the gut microbiota in a specific population and determine what factors affect its composition. Here, we studied the bacterial gut microbiota, and its association with environmental factors, of Indonesian schoolchildren living in urban Makassar.

## 2. Methods

### 2.1. Study Design and Ethics Approval

This study was a cross-sectional study involving Indonesian schoolchildren living in an urban area of Makassar. Children from two public schools, which were distinct in socioeconomic level, were included in this study. The study was approved by the ethics committee for medical research of the Faculty of Medicine, Universitas Hasanuddin, Indonesia (approval number: 1504/H04.8.4.5.31/PP36-KOMETIK/2016). Written, signed, and dated informed consent was obtained from the parents or guardian of each child prior to the study.

### 2.2. Study Population and Data Collection

One hundred and forty children were recruited from one high (*n* = 74) and one low SES (*n* = 66) school. The high SES school is located in the city centre and is considered of high status, with a majority of the parents working as high-skilled workers or professionals with higher education. Meanwhile, the low SES school is located near a landfill and port area, where most of the parents are low-educated and work on low-skilled labour jobs.

A standardised questionnaire was used to gather demographic information of the children, including age and sex. To determine the nutritional status, anthropometric measurement was performed. To assess the intestinal parasitic infection and gut microbiota composition, stool samples were collected using a stool container with enclosed spoon (Sarstedt AG&Co.KG, Nümbrecht, Germany). As soon as samples were collected by research staff, samples were stored inside an ice bag and transported to the Laboratory of Parasitology Department at Hasanuddin University to be aliquoted and stored at −80 °C. The children’s characteristics are presented in Table 1.

### 2.3. Anthropometric Measurements

The height and weight of the participant were measured using a portable stadiometer (SECA GmbH & Co., Hamburg, Germany) and digital scale (GEA, Megapratama Medikalindo, Indonesia). The weighing scale was calibrated using standardised weight as part of routine care. BMI-for-age (zBMI) was calculated according to the World Health Organization (WHO) references value to determine the nutritional status of the participants [14]. Children were categorised as thinness if zBMI < −2 SD, normal if zBMI was between −2 SD to 1 SD, and overweight/obese if zBMI > 1SD.

### 2.4. Parasitological Examination

A single-slide of each freshly-collected stool sample was examined by the Katokatz methods for the detection of soil transmitted helminths eggs. DNA was extracted from cryopreserved stool for intestinal protozoa identification using a QIAamp Spin Columns/Mini Kit (Qiagen, Germany) [15]. In each sample, a fixed amount of Phocine Herpes Virus 1 was included within the isolation lysis buffer as an internal control [16]. A panel of multiplex real-time Polymerase Chain Reaction (PCR) was used to detect and quantify intestinal protozoa, targeting *Entamoeba histolytica, Dientamoeba fragilis, Giardia lamblia*, and *Cryptosporidium parvum.* The procedure has been described elsewhere [15,17].

### 2.5. Microbiota Analysis

DNA was extracted from approximately 0.1 g of cryopreserved stool using the Quick-DNA™ Fecal/Soil Microbe Miniprep Kit (ZymoResearch, CA, USA) according to manufacturer instructions. Quality control, library preparation, and 16S rRNA gene amplicon sequencing were performed by BaseClear (Leiden, The Netherlands), targeting the V3-V4 region (F: CCTACGGGNGGCWGCAG, R: GACTACHVGGGTATCTAATCC), and using the Illumina MiSeq platform (300 bp, paired-end, Illumina, CA, USA). Raw sequencing data are available in the European Nucleotide Archive (https://www.ebi.ac.uk/ena) under study accession PRJEB38465.

Read filtering, operational taxonomic unit (OTU)-picking, and taxonomic assignment were performed using the NG-Tax 0.4 pipeline with the following settings: forward and reverse read length of 120, ratio OTU abundance of 2.0, classify ratio of 0.9, minimum threshold of 1 × 10^−7^, identity level of 100%, and an error correction of 98.5, using the Silva_132_SSU Ref database [18,19]. The obtained OTU-table was filtered for OTUs with a number of sequences less than 0.005% of the total number of sequences [20].

Six positive controls (ZymoBiomics Microbial Community Standard, ZymoResearch, Leiden, The Netherlands) and six negative controls (empty extractions) were taken along from DNA extraction onwards, meaning one control per DNA extraction kit. In addition, four positive controls (ZymoBiomics Microbial Community DNA Standard, ZymoResearch, Leiden, The Netherlands) were taken along from Quality Control onwards, meaning one control per sequencing run. The eight bacterial species present in the included positive controls were all identified. The relative abundance of these species was on average 1.04 ± 0.20 and 1.06 ± 0.30-fold different from the theoretical abundances for the sequencing control and DNA extraction control, respectively. This indicates that minor variation is induced by the sequencing and DNA extraction procedures. Four additional bacterial taxa were identified in the positive controls, namely *Collinsella*, *Bifidobacterium*, *Enterobacteriaceae*, and *Catenibacterium*, but their relative abundance only accounted for 0.017 ± 0.013% of total relative abundance. The included negative controls resulted in non-quantifiable DNA concentrations using the Qubit™ dsDNA HS Assay Kit (Thermo Fisher, Landsmeer, The Netherlands) on a Qubit 3.0 Fluorometer (Invitrogen, Breda, The Netherlands), but they were sequenced nevertheless, resulting in approximately ten times less reads than the stool samples, with *Delftia* and *Staphylococcus* as the most abundant contaminants.

### 2.6. Statistical Analysis

Prevalence rates were calculated as percentage of collected data and compared between schools using the Pearson chi-square test. Comparisons between groups for continuous data were analysed using the Student *t*-test for normally distributed data, and using the Mann-Whitney-U test if the data were not normally distributed. This analysis was performed using IBM SPPS Statistics version 25. (IBM-SPSS Inc., Chicago, IL, USA).

Statistical analysis and data visualisation for microbiota data were performed in R (v3.6.1) using the packages phyloseq (v1.30.0) [21], vegan (v2.5-6) [22], ggplot2 (v3.2.1) [23], DESeq2 (v1.22.2) [24], and microbiome (v1.8.0) [25]. All analyses were performed on genus-level, except for alpha-diversity measures (Shannon diversity and observed richness). For differential abundance testing by DESeq2, genera present in less than 25% of the samples were removed to minimise zero-variance errors and spurious significance. Outcomes were considered significant when the Benjamini–Hochberg corrected *p*-value was ≤ 0.05. Bacterial taxa were considered part of the core microbiota when present in 95% of the samples from the specified group. Permutational multivariate analysis of variance (PERMANOVA) was performed using the adonis function with 999 permutations and Bray–Curtis dissimilarity to determine associations between microbiota composition and clinical variables. Outcomes were considered significant when the *p*-value was ≤ 0.05.

## 3. Results

### 3.1. The Bacterial Gut Microbiota of Indonesian Schoolchildren

To obtain a comprehensive overview of the bacterial gut microbiota of Indonesian school children, microbiota composition, as well as factors influencing their microbiota composition, were determined. The core microbiota consisted of *Bifidobacterium*, *Collinsella*, and multiple members of the *Lachnospiraceae* and *Ruminicoccaceae* families (Table 2), together constituting an average relative abundance of 56.3 ± 19.8%. With an average relative abundance of 23.5% and 13.4%, *Bifidobacterium* and *Collinsella* were the most abundant members of the bacterial community (Table 3). However, their relative abundance varied greatly between children, ranging from 0.8 to 78.6% and 0.0 to 65.2%, respectively (Table 3). Large variation in relative abundance was also observed for the other most abundant bacterial taxa (Table 3). Variation in overall microbiota composition was significantly associated with socioeconomic status, as determined by the Bray–Curtis dissimilarity-based multivariate analysis (PERMANOVA; R2 = 0.049; *p* = 0.001).

**Figure 1:**
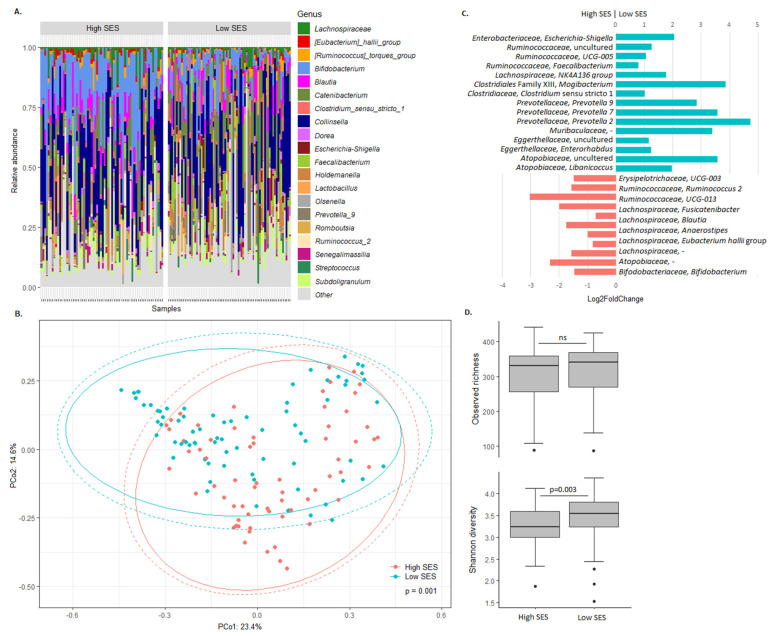
Association between the bacterial gut microbiota and SES. (**A**) Taxonomic profiles of the gut microbiota of high and low SES children. The 20 most abundant bacterial taxa are shown. Relative abundance of all other taxa is summed and labelled as ‘other.’ (**B**) The Bray–Curtis dissimilarity-based principal coordinate analysis plot labelled according to SES. Ellipses indicate confidence assuming a multivariate t-distribution (solid line) or a multivariate normal distribution (dotted line). (**C**) Differential abundance of bacterial taxa between high and low SES children. Taxa with Benjamini–Hochberg corrected *p*-value below 0.05 are shown. (**D**) Alpha diversity in high and low SES children. Boxplots show the median, 25th and 75th percentiles, and minimal and maximal values with the exception of outliers (black circles).

To explore the relation between SES and the bacterial gut microbiota, microbiota composition and bacterial richness/diversity were compared between high and low SES children (Figure 1). Taxonomic profiles and principal coordinate analysis confirmed the difference, but also showed the similarity, in overall microbiota composition between high and low SES children (Figure 1A,B). Differential abundance analysis revealed that, among others, *Bifidobacterium*, *Lactobacillus*, and various *Lachnospiraceae* and *Ruminococcaeae* members were more abundant in high SES children, while *Prevotella*, *Mogibacterium*, *Escherichia-Shigella*, and other members of the *Lachnospiraceae* and *Ruminococcaceae* families were more abundant in low SES children (Figure 1C). While bacterial richness was comparable, diversity was significantly higher in low SES children (Figure 1D).

### 3.2. The Bacterial Gut Microbiota of High SES Indonesian Schoolchildren

To obtain a comprehensive overview of the bacterial gut microbiota of high SES children (*n* = 66), microbiota composition, as well as factors influencing their microbiota composition, were determined. The core microbiota of high SES children consisted of *Bifidobacterium*, *Collinsella*, *Clostridium* sensu stricto 1, *Romboutsia*, and multiple members of the *Lachnospiraceae* and *Ruminicoccaceae* families (Table 2), constituting an average relative abundance of 65.6 ± 15.2%, with *Bifidobacterium* and *Collinsella* being most abundant (Table 3). Variation in overall microbiota composition was significantly associated with nutritional status (zBMI; PERMANOVA; R2 = 0.036; *p* = 0.011).

**Figure 2:**
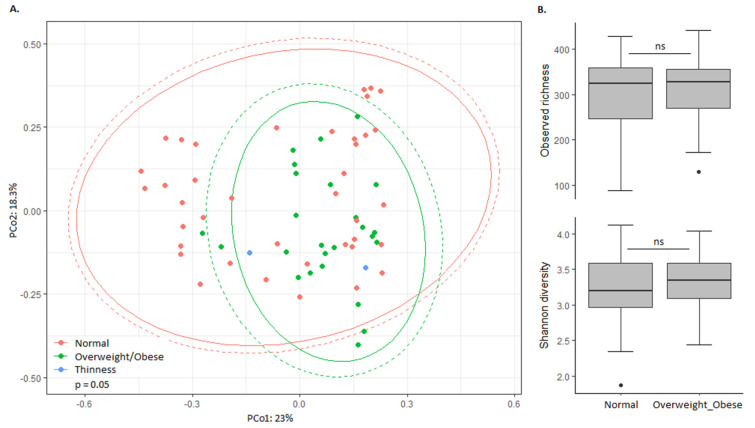
Association between the bacterial gut microbiota and nutritional status in high SES children. (**A**) The Bray–Curtis dissimilarity-based principal coordinate analysis plot labelled according to nutritional status. Ellipses indicate confidence assuming a multivariate t-distribution (solid line) or a multivariate normal distribution (dotted line). (**B**) Alpha diversity in high SES children with normal weight and overweight/obesity. Boxplots show the median, 25th and 75th percentiles, and minimal and maximal values with the exception of outliers (black circles).

To explore the relation between nutritional status and the bacterial gut microbiota, microbiota composition and bacterial richness/diversity were compared between normal weight and overweight/obese high SES children (Figure 2). Despite the association of zBMI with overall microbiota composition (Figure 2A), bacterial richness and diversity were similar between normal weight and overweight/obese children (Figure 2B), and only *Eggerthella* was significantly differentially abundant (Log2FoldChange = −3.49; padj = 0.005), with higher relative abundance in normal weight children.

### 3.3. The Bacterial Gut Microbiota of Low SES Indonesian Schoolchildren

To obtain a comprehensive overview of the bacterial gut microbiota of low SES children (*n* = 74), microbiota composition, as well as factors influencing their microbiota composition, were determined. The core microbiota of low SES children consisted of *Bifidobacterium*, *Collinsella*, *Senegalimassilia*, *Holdemanella*, and multiple members of the *Lachnospiraceae* and *Ruminicoccaceae* families (Table 2), constituting an average relative abundance of 54.4 ± 20.8%, with *Collinsella* being the most abundant (Table 3). None of the included variables were significantly associated with overall microbiota composition in low SES children. Nevertheless, the high prevalence of helminth infection (60.8%) and protozoa infection (65.8%) in this group prompted us to study their association with microbiota composition.

**Figure 3:**
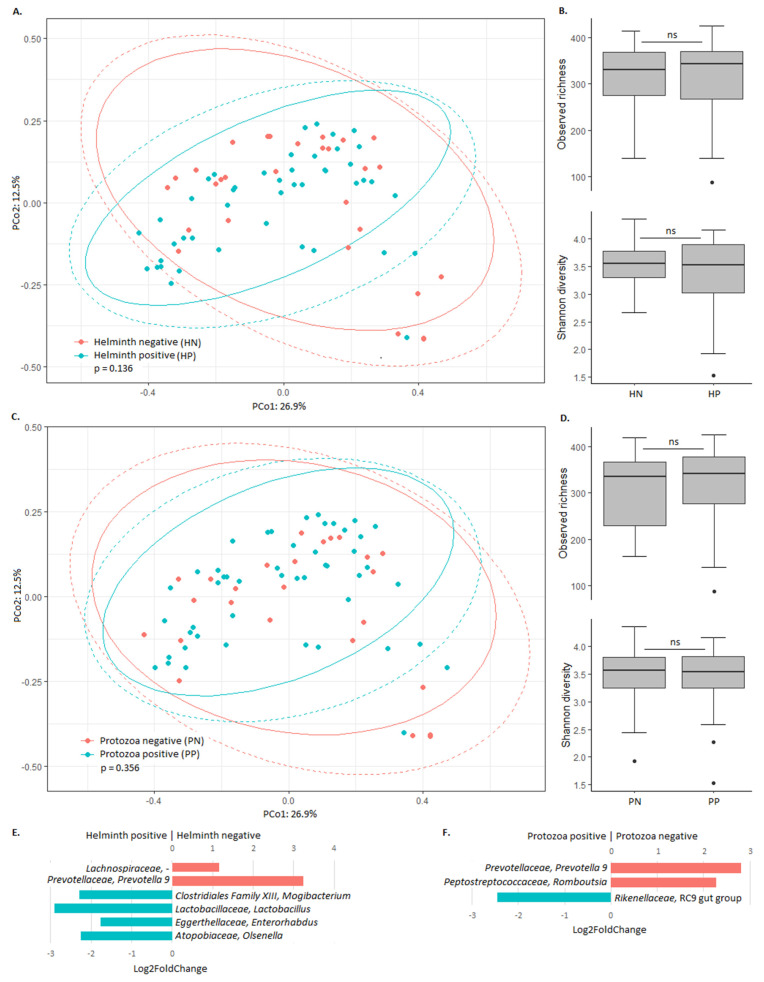
Association between the bacterial gut microbiota and helminth and protozoa infection in low SES children. (**A**) The Bray–Curtis dissimilarity-based principal coordinate analysis plot labelled according to helminth infection. Ellipses indicate confidence assuming a multivariate t-distribution (solid line) or a multivariate normal distribution (dotted line). (**B**) Alpha diversity in low SES children that are helminth negative (HN) and helminth positive (HP). Boxplots show the median, 25th and 75th percentiles, and minimal and maximal values with the exception of outliers (black circles). (**C**) The Bray–Curtis dissimilarity-based principal coordinate analysis plot labelled according to protozoa infection. Ellipses indicate confidence assuming a multivariate t-distribution (solid line) or a multivariate normal distribution (dotted line). (**D**) Alpha diversity in low SES children that are protozoa negative (PN) and protozoa positive (PP). Boxplots show the median, 25th and 75th percentiles, and minimal and maximal values with the exception of outliers (black circles). (**E**) Differential abundance of bacterial taxa between helminth positive and helminth negative low SES children. Taxa with Benjamini–Hochberg corrected *p*-value below 0.05 are shown. (**F**) Differential abundance of bacterial taxa between protozoa positive and protozoa negative low SES children. Taxa with Benjamini–Hochberg corrected *p*-value below 0.05 are shown.

To explore the relation between helminth or protozoa infection and the bacterial gut microbiota, microbiota composition and bacterial richness/diversity were compared between low SES children with and without these infections (Figure 3). Principal coordinate analysis confirmed that overall microbiota composition was not associated with helminth or protozoa status (Figure 3A,C). In addition, the bacterial richness and diversity were similar between children with and without helminth or protozoa infection (Figure 3B,D). Despite these commonalities, various bacterial taxa were differentially abundant. Relative abundance of *Olsenella*, *Enterorhabdus*, *Lactobacillus*, and *Mogibacterium* was higher in helminth infected children, whereas the relative abundance of *Prevotella* and unclassified *Lachnospiraceae* was higher in children without helminth infection (Figure 3E). In addition, the relative abundance of the *Rikenellaceae* RC9 gut group was higher in protozoa infected children, whereas the relative abundance of *Prevotella* and *Romboutsia* was higher in children without protozoa infection (Figure 3F).

## 4. Discussion

Gut microbiota composition is largely influenced by environmental factors. In this study, socioeconomic status was the main factor associated with bacterial gut microbiota composition of Indonesian schoolchildren. Bacterial diversity was lower in children from high socioeconomic status, and their microbiota contains a higher relative abundance of *Bifidobacterium* and *Lactobacillus*, while containing a lower relative abundance of *Prevotella* and *Escherichia-Shigella*, among others. The gut microbiome plays a key role in host metabolism and immune functioning, and microbial dysbiosis during early life has been associated with the development of several diseases in later life, including atopy, obesity, chronic inflammatory conditions, and infections [26]. Alterations in microbiota composition as observed in our study may, therefore, contribute to the inequalities in health status and life expectancy of children from low and high socioeconomic status. Vice versa, early life exposures are likely to be affected by socioeconomic status-associated differences in lifestyle, education and healthcare, all which influence gut microbiota composition and may explain the observed differences in gut microbiota composition between low and high SES children.

Similar to our study, Chong CW et al. showed lower microbial diversity in wealthier children as compared to economically deprived children living in the same rural area in Malaysia [27]. Such differences in microbiota structure most likely result from lifestyle differences. Children from low SES are expected to have higher exposure to microorganisms and parasites [28]. In addition, diet is considered a main driver of gut microbiota composition [12]. As such, observed differences in microbiota composition between high and low SES children may be reflecting differences in their diet. High SES children are more likely to be exposed to high-fat diet products as compared to low SES children [29]. The high abundance of *Bifidobacterium* and *Lactobacillus* in high SES children could be associated with higher probiotics intake [30]. High SES children consume dairy products regularly, and probiotic drinks were available in the canteen of the high SES school, but not in the low SES school. *Prevotella*, observed to be of higher relative abundance in low SES children, has been associated with a higher intake of vegetables and fibre [31]. However, the influence of diet on microbiota composition remains speculative herein, as food intake was not determined in our study.

In high SES children, *Bifidobacterium*, *Collinsella*, *Clostridium* sensu stricto 1, *Romboutsia*, and multiple members of the *Lachospiraceae* and *Ruminicoccaceae* families were prevalent. Overall, the microbiota composition was mostly associated with nutritional status, and except for the differential abundance of *Eggerthella*, relative abundance of individual bacterial taxa did not significantly differ between normal weight and overweight/obese children, nor did bacterial richness and diversity. A recent study investigating Asian children from five different countries also found *Bifidobacterium* as the most abundant bacterium, among others [32]. In addition, nutritional status has previously been associated with microbiota composition in Mexican children [33]. It is important to realise that these differences may be a result of underlying variations in diet and environmental, geographical, demographic, and clinical factors. After correction for such factors, however, a significant association between microbiota and BMI has been shown in children who were part of the American Gut Project [34].

In low SES children, the core microbiota consisted of *Bifidobacterium*, *Collinsella*, *Senegalimassilia*, *Holdemanella*, and multiple members of the *Lachnospiraceae* and *Ruminicoccaceae* families. Helminth infection was prevalent in low SES children, and was positively associated with *Olsenella, Enterorhabdus, Lactobacillus*, and *Mogibacterium* abundance, while it was negatively associated with the relative abundance of *Prevotella*. In addition, protozoa infection was prevalent in low SES children, and was also negatively associated with the relative abundance of *Prevotella*. We observed no apparent association between helminth or protozoa infection and microbiota diversity. Recently, there has been much interest in the relationship between helminth infection and the gut microbiota as they shared the same niche in the human body. Infection with intestinal helminths can impact the intestinal microbiome, with important consequences for each player in the tripartite relationship between the host, helminth, and the microbiota [35]. In a previous study conducted in rural Flores, Indonesia, where helminth infection is highly prevalent, no association between helminth infection and gut microbiota diversity was observed [13]. In contrast to our study, increased diversity of gut microbiota has been observed in helminth colonised people in rural Malaysia [36]. Regarding protozoa, a previous study conducted in rural Cameroon showed higher alpha diversity in people infected by *Entamoeba* (*E. histolytica*, *E. dispar*, or both). Furthermore, *Entamoeba* presence was also associated with gut microbiota composition [37]. Discrepancies in findings most likely result from differences in the included population, population size, and the specific intestinal parasite species someone is infected with. Further studies are needed to elucidate the complex “three-way” relationship between intestinal parasites, microbiota, and the human host.

## 5. Conclusions

We demonstrated that Indonesian schoolchildren living in urban Makassar share a core microbiota consisting of *Bifidobacterium*, *Collinsella*, and multiple members of the *Lachnospiraceae* and *Ruminicoccaceae* families, but that their microbiota varies in the diversity and relative abundance of specific bacterial taxa depending on socioeconomic status, nutritional status, and helminth and protozoa infection. These findings contribute to increased understanding of the environmental factors shaping gut microbiota composition in Indonesian schoolchildren.

## Figures and Tables

**Table 1:** Characteristics of study populations.

Characteristics	All Children (*n* = 140)	High SES Children (*n* = 74)	Low SES Children (*n* = 66)	*p*-Value
Age, years, mean ± SD	10.33 ± 0.85	10.40 ± 0.56	10.25 ± 1.04	0.294
Female: n (%)	85 (60.7)	41 (62.1)	44 (59.5)	0.748
zBMI, mean ± SD	−0.31 ± 1.46	0.33 ± 1.5	−0.89 ± 1.14	<0.001
Nutritional status (WHO): n (%)				<0.001
Thinness	13 (9.3)	2 (3.0)	11 (14.9)	
Normal	99 (70.7)	39 (59.1)	60 (81.1)	
Overweight and Obese	28 (20.0)	25 (37.9)	3 (4.1)	
Intestinal parasitic infection: n (%)				
Any helminth	47 (33.6)	2 (3.0)	45 (60.8)	<0.001
*Ascaris lumbricoides*	34 (24.3)	0	34 (45.9)	
*Trichuris trichiura*	25 (17.9)	2 (3.0)	23 (31.1)	<0.001
*Hymenolepis diminuta*	1 (0.7)	0	1 (1.4)	
Any protozoa	66 (47.1)	18 (27.3)	48 (65.8)	<0.001
*Entamoeba histolytica,*	14 (10.0)	0	14 (19.2)	
*Dientamoeba fragilis*	30 (21.4)	10 (15.2)	20 (27.4)	0.080
*Giardia lamblia*	39 (27.9)	9 (13.6)	30 (41.1)	<0.001
*Cryptosporidium parvum*	2 (1.4)	0	2 (2.7)	

The number of positives (n) of the total population examined (*n* = 140). Statistical testing was performed using the student t-test for continuous variables and using the chi-square test for categorical variables. SD: standard deviation, SES: socioeconomic status.

**Table 2:** Core microbiota in all, high SES, and low SES children.

Family	Genus	All Children	High SES Children	Low SES Children
*Bifidobacteriaceae*	*Bifidobacterium*	x	x	x
*Coriobacteriaceae*	*Collinsella*	x	x	x
*Ruminococcaceae*	*Faecalibacterium*	x	x	x
*Ruminococcaceae*	*Subdoligranulum*	x	x	x
*Lachnospiraceae*	*-*	x	x	x
*Lachnospiraceae*	*Blautia*	x	x	x
*Lachnospiraceae*	*Dorea*	x	x	x
*Lachnospiraceae*	*Fusicatenibacter*	x	x	x
*Lachnospiraceae*	*Eubacterium hallii* group	x	x	x
*Lachnospiraceae*	*Ruminococcus torques* group	x		x
*Peptostreptococcaceae*	*Romboutsia*		x	
*Clostridiaceae 1*	*Clostridium* sensu stricto 1		x	
*Erysipelotrichaceae*	*Holdemanella*			x
*Eggerthellaceae*	*Senegalimassilia*			x

Core taxa (x) are considered those present in 95% of the samples from the specified group.

**Table 3:** The ten most abundant bacterial taxa in all, high SES, and low SES children.

	Family	Genus	Average ± SD	Min–Max
**All children**	*Coriobacteriaceae*	*Collinsella*	0.235 ± 0.188	0.009–0.786
	*Bifidobacteriaceae*	*Bifidobacterium*	0.134 ± 0.139	0.000–0.652
	*Erysipelotrichidae*	*Catenibacterium*	0.077 ± 0.090	0.000–0.425
	*Lachnospiraceae*	*Blautia*	0.049 ± 0.047	0.001–0.208
	*Prevotellaceae*	*Prevotella 9*	0.044 ± 0.118	0.000–0.638
	*Ruminococcaceae*	*Faecalibacterium*	0.043 ± 0.056	0.000–0.322
	*Erysipelotrichaceae*	*Holdemanella*	0.031 ± 0.055	0.000–0.337
	*Ruminococcaceae*	*Subdoligranulum*	0.030 ± 0.035	0.000–0.224
	*Lachnospiraceae*	*-*	0.024 ± 0.038	0.000–0.255
	*Coriobacteriaceae*	*Olsenella*	0.024 ± 0.077	0.000–0.729
**High SES children**	*Coriobacteriaceae*	*Collinsella*	0.246 ± 0.185	0.013 ± 0.786
	*Bifidobacteriaceae*	*Bifidobacterium*	0.198 ± 0.152	0.004 ± 0.652
	*Erysipelotrichidae*	*Catenibacterium*	0.065 ± 0.082	0.000 ± 0.416
	*Lachnospiraceae*	*Blautia*	0.055 ± 0.050	0.001 ± 0.208
	*Ruminococcaceae*	*Subdoligranulum*	0.033 ± 0.038	0.000 ± 0.224
	*Lachnospiraceae*	*-*	0.030 ± 0.047	0.000 ± 0.255
	*Ruminococcaceae*	*Faecalibacterium*	0.029 ± 0.044	0.000 ± 0.266
	*Ruminococcaceae*	*Ruminococcus 2*	0.029 ± 0.059	0.000 ± 0.292
	*Erysipelotrichaceae*	*Holdemanella*	0.028 ± 0.059	0.000 ± 0.337
	*Coriobacteriaceae*	*Olsenella*	0.025 ± 0.064	0.000 ± 0.456
**Low SES children**	*Coriobacteriaceae*	*Collinsella*	0.225 ± 0.190	0.009 ± 0.737
	*Erysipelotrichidae*	*Catenibacterium*	0.088 ± 0.095	0.000 ± 0.425
	*Bifidobacteriaceae*	*Bifidobacterium*	0.077 ± 0.095	0.000 ± 0.403
	*Prevotellaceae*	*Prevotella 9*	0.071 ± 0.150	0.000 ± 0.638
	*Ruminococcaceae*	*Faecalibacterium*	0.055 ± 0.062	0.000 ± 0.322
	*Lachnospiraceae*	*Blautia*	0.043 ± 0.043	0.001 ± 0.170
	*Erysipelotrichaceae*	*Holdemanella*	0.035 ± 0.051	0.000 ± 0.267
	*Ruminococcaceae*	*Subdoligranulum*	0.029 ± 0.031	0.001 ± 0.183
	*Coriobacteriaceae*	*Olsenella*	0.023 ± 0.087	0.000 ± 0.729
	*Lachnospiraceae*	*-*	0.019 ± 0.026	0.000 ± 0.119

Relative abundances (average, min, max) are presented as a fraction of the total relative abundance. SD: standard deviation, SES: socioeconomic status.
